# Assessing capacities and resilience of health services during the COVID-19 pandemic: Lessons learned from use of rapid key informant surveys

**DOI:** 10.3389/fpubh.2023.1102507

**Published:** 2023-02-13

**Authors:** Briana Rivas-Morello, Dirk Horemans, Kavitha Viswanathan, Chelsea Taylor, Andrea Blanchard, Humphrey Karamagi, Benson Droti, Regina Titi-Ofei, Laetitia Ouedraogo Nikiema, Moussa Traore, Hillary Kipruto, Amalia del Riego, Natalia Houghton, Hassan Salah, Deena Alasfoor, Henry Doctor, Ardita Tahirukaj, Florian Tille, Tomas Zapata, Kathryn O'Neill

**Affiliations:** ^1^Health Services Performance Assessment Unit, Integrated Health Services Department, World Health Organization, Geneva, Switzerland; ^2^Institute of Global Public Health, Department of Community Health Services, Rady Faculty of Health Sciences, University of Manitoba, Winnipeg, MB, Canada; ^3^Data Analytics and Knowledge Management, Office of the Regional Director, Regional Office for Africa, World Health Organization, Brazzaville, Republic of Congo; ^4^Health Information System Unit, Universal Health Coverage/Life Course Cluster, Regional Office for Africa, World Health Organization, Brazzaville, Republic of Congo; ^5^Health Services and Access Unit, Health Systems and Services Department, Pan American Health Organization/Regional Office for the Americas, World Health Organization, Washington, DC, United States; ^6^Access to Health Services Unit, Department for Universal Health Coverage/Health Systems, Regional Office for the Eastern Mediterranean, World Health Organization, Cairo, Egypt; ^7^Information Systems for Health Unit, Department for Science, Information and Dissemination, Regional Office for the Eastern Mediterranean, World Health Organization, Cairo, Egypt; ^8^Emergency Operations Unit, WHO Health Emergencies Programme, Regional Office for Europe, World Health Organization, Copenhagen, Denmark; ^9^European Observatory on Health Systems and Policies, Regional Office for Europe, World Health Organization, Copenhagen, Denmark; ^10^Health Workforce and Service Delivery Unit, Division of Country Health Policies and Systems, Regional Office for Europe, World Health Organization, Copenhagen, Denmark

**Keywords:** COVID-19, resilience, recovery, health service capacities, key informant surveys, facility and community surveys

## Abstract

**This article is part of the Research Topic:**

‘Health Systems Recovery in the Context of COVID-19 and Protracted Conflict.’

**Problem:**

Many countries lacked rapid and nimble data systems to track health service capacities to respond to COVID-19. They struggled to assess and monitor rapidly evolving service disruptions, health workforce capacities, health products availability, community needs and perspectives, and mitigation responses to maintain essential health services.

**Method:**

Building on established methodologies, the World Health Organization developed a suite of methods and tools to support countries to rapidly fill data gaps and guide decision-making during COVID-19. The tools included: (1) a national “pulse” survey on service disruptions and bottlenecks; (2) a phone-based facility survey on frontline service capacities; and (3) a phone-based community survey on demand-side challenges and health needs.

**Use:**

Three national pulse surveys revealed persisting service disruptions throughout 2020–2021 (97 countries responded to all three rounds). Results guided mitigation strategies and operational plans at country level, and informed investments and delivery of essential supplies at global level. Facility and community surveys in 22 countries found similar disruptions and limited frontline service capacities at a more granular level. Findings informed key actions to improve service delivery and responsiveness from local to national levels.

**Lessons learned:**

The rapid key informant surveys provided a low-resource way to collect action-oriented health services data to inform response and recovery from local to global levels. The approach fostered country ownership, stronger data capacities, and integration into operational planning. The surveys are being evaluated to inform integration into country data systems to bolster routine health services monitoring and serve as health services alert functions for the future.

## Introduction

The maintenance of essential health services during the corona virus disease of 2019 (COVID-19) pandemic has been critical, as disruptions to essential health services—including for health promotion, disease prevention, diagnosis, treatment, rehabilitation, and palliation—may lead to even greater adverse health outcomes than the pandemic itself, especially in vulnerable populations (1–7). However, throughout the pandemic, many countries have faced complex challenges that required accurate and timely data on facility capacities, service utilization, and community needs and preferences to inform the development of action plans and strategies to respond to COVID-19 while maintaining safe delivery of care.

Country health information systems generally comprise of many different data sources, including population-based surveys, civil registration and vital statistics systems, facility assessments, routine health information systems (RHIS), health workforce information systems, and financial information systems among others. Even before the pandemic, many countries faced pre-existing weaknesses in these systems, including around data access, availability, quality, timeliness and use. The World Health Organization's (WHO) 2021 Global report on health data systems and capacities showed that 65% of 133 countries had only moderate or lower capacities for availability of health services data (8). The COVID-19 pandemic placed even greater strains on country data systems globally.

Whilst there are well-established survey methodologies and routine data systems used by governments to monitor different aspects of service delivery (9–26), most were not designed to provide rapid and comprehensive evidence on dynamic aspects of service capacities and delivery needed to inform the immediate adaptation of service provision during the pandemic. They were also not devised to monitor the implementation of mitigation strategies, or track longer-term health service recovery over time.

To rapidly bolster and supplement country data systems and capacities, a suite of rapid methods and tools was developed to track and monitor health service readiness, resilience and responsiveness during the COVID-19 pandemic and for future health crises.

This work was led by WHO in collaboration with Member States, and with contributions from global partners of the Access to COVID-19 Tools Accelerator^1^, including the United Nations Children's Fund, the World Bank and Global Financing Facility, Gavi the Vaccine Alliance, and The Global Fund to Fight AIDS, Tuberculosis, and Malaria (27).

This paper provides an overview of the implemented methods and tools, introduces illustrative results of the types of findings that were generated and their use, and identifies early lessons learned. Further publications are forthcoming on additional in-depth analyses of country data, country experiences on data use, and implications for ensuring sustainable health services surveillance and monitoring systems for the future.

## Methods

A suite of methods and tools was designed to complement existing country data systems and bolster capacities to monitor health service readiness, resilience and responsiveness, with an emphasis on supporting the continuity of essential health services during the COVID-19 pandemic. The tools were designed for implementation on a regular basis, in order to track trends in health service recovery and fluctuating service capacities over time. They contributed to a broader approach that aimed to strengthen country data capacities and platforms for tracking health services during the pandemic and into recovery.

The methods and tools were harmonized to supplement each other and support use of data at different levels of the health system. The suite included: (1) a national key informant “pulse” survey on continuity of essential health services that was administered to all countries; (2) rapid phone-based surveys in a sample of frontline health facilities on service capacities; and (3) rapid phone-based surveys in a sample of community representatives (most often, community providers) to provide demand-side understanding of the evolving health challenges and needs faced by communities.

The facility and community surveys were particularly designed to augment data from existing RHIS, national surveillance systems and other administrative sources. Many countries have well-established RHIS to provide regular information on service utilization and certain aspects of capacities. As noted previously, however, the use of RHIS data is often hampered by timeliness and quality issues—which were further exacerbated by the pandemic. Moreover, RHIS were not designed to capture qualitative details on the extent of disruptions, reasons for disruptions, usefulness of different mitigation strategies, or dynamic details of service capacities during a health crisis.

### National key informant survey on continuity of essential health services

WHO has conducted three rounds of the “pulse” survey on continuity of essential health services during the COVID-19 pandemic. In the absence of other globally comparable data, the survey provided rapid insights from national level country key informants into the extent of impact of the COVID-19 pandemic on health systems and essential health services, and priority needs in terms of resources and support against a quickly changing context (28–30).

The first survey was implemented during May-September 2020 (28), the second survey was implemented during January-March 2021 (29), and the third survey was implemented during November-December 2021 (30). The next pulse survey is planned for October-December 2022. The results in this paper are presented for 97 countries that completed at least one survey section for all three rounds of the pulse survey. This includes 36 countries in the African region, 21 countries in the Americas region, 17 countries in the Eastern Mediterranean region, 10 countries in the European region, eight countries in the Southeast Asia region, and five countries in the Western-pacific region^2^.

#### Content

The pulse survey was designed in modular survey sections targeting different national level key informants in each country. It included a cross-cutting section covering governance aspects, disruptions to service delivery settings (including primary, community, emergency, critical, operative, rehabilitative, and palliative care), mitigation strategies, and main health system bottlenecks and needs.

It also included in-depth sections to track disruptions across tracer health service areas, including: sexual, reproductive, maternal, newborn, child and adolescent health; nutrition; care for older people; immunization; human immunodeficiency virus (HIV) and hepatitis; tuberculosis; malaria; neglected tropical diseases (NTD); non-communicable diseases, and mental, neurological and substance use disorders. The survey integrated and built on targeted WHO surveys that were disseminated early in the pandemic on specific tracer service disruptions (31–33).

Each survey asked key informants to consider the situation in countries during a specific period of time: 3 months prior to survey response for the first two surveys (28, 29), and 6 months prior to survey response for the third survey (30).

#### Implementation

The pulse survey was distributed to Ministries of Health in all countries. It was disseminated through WHO Regional Offices and WHO Country Offices using a secure web-based questionnaire in LimeSurvey software (34). The questionnaire was available in Arabic, Chinese, English, French, Portuguese, Russian and Spanish. Two or more reminders to complete they survey were sent were to all countries.

Respondents included health policy advisors, directors of health services, systems, or programmes, monitoring and evaluation focal points, public health officers and/or incident management team focal points within Ministries of Health and/or WHO Country Offices. The exact process for survey completion was flexible and varied by country, ranging from independent completion of sections by different key informants, to coordinated completion of sections based on collaborative key informant discussions. Completed country profiles were disseminated to countries through WHO regional offices.

### Frontline health service capacity surveys (health facility and community surveys)

Since September 2020, WHO has supported a subset of countries that expressed country demand to implement rapid, high-frequency phone-based surveys to gain more granular insights into frontline health service capacity and delivery challenges faced at facility and community levels. The surveys aimed to enable more safe and real-time data collection, analysis and use throughout the rapidly evolving pandemic context.

They were designed for modular administration in hospitals, primary care facilities, and communities. Countries could tailor and implement different combinations of modules for either one-time or recurrent use based on context, priorities, resources, and need at different points of the pandemic (35).

This paper focuses on results from 22 countries that conducted at least one facility or community survey between December 2020 and March 2022. This includes 12 countries in the African region, five countries in the Americas region, three countries in the Eastern Mediterranean region, and two countries in the European region (see Annex 1 for details). Each country implemented 1–3 survey rounds.

#### Content

##### Health facility survey

The facility survey included two core modules to support countries to assess and track:

a. COVID-19 case management capacities, with an emphasis on availability of therapeutics, diagnostics, oxygen, personal protective equipment (PPE), vaccines, and vaccine readiness (36).
b. Continuity of essential health services, and facility and workforce capacities to maintain the safe provision of care (37).

##### Community survey

The community survey module focused on measuring community needs and perceptions, changes in care-seeking behaviors, and barriers to accessing care during the pandemic (38).

Further details on the three tools are presented in Annex 2.

Of note, while the above modules are the focus of this paper, the suite included additional facility checklists and inventory tools on hospital readiness (39, 40), biomedical equipment availability (41, 42), safe environment measures (43), and infection prevention and control (44). Countries could consider use of these modules for in-depth assessments as needed.

#### Methodology and implementation

The recommended methodology for the health facility and community surveys was phone-based interviews with facility managers and/or community representatives in a sample of facilities and communities. Responses were input into an online data collection instrument using a secure web-based questionnaire (34).

For the facility survey, the methodology recommended to randomly select 80–100 health facilities through a stratified sampling approach using a master facility list. For the community survey, it was recommended to select one community representative from the catchment areas of each primary care facility in the sample^3^.

To track changes and trends throughout the rapidly changing COVID-19 context, the recommended frequency was to conduct a facility survey 2–4 times per year, with the supporting community survey implemented at intervals.

A package of implementation guidance and template materials was developed to enable rapid implementation and ultimate absorption into country data systems (45). It included standard data collection instruments using LimeSurvey (34), standard analysis codes in Stata (46) and R (47), automated outputs and visualizations in Excel (48), and template dashboards using ReactJS (49), and Kendo UI (50).

#### Key analyses

Analyses presented in this paper are based on data from the most recent survey round in each country, which ranging from January 2021 to March 2022. This includes data from 498 higher-level facilities (mainly hospitals) in 18 countries, 2,377 lower-level facilities (mainly primary care facilities) in 21 countries, and 1,277 community representatives (mainly community health workers) in 17 countries.

It covers descriptive analyses on changes in service volumes, reasons for disruptions, mitigation measures taken by facility management, community perspectives and needs, and availability of key health resources in hospitals and primary care settings. Definitions for availability of key health products differ by level of care as follows:

- Percentage of facilities with all tracer PPE items available for all staff Items include gloves and medical/surgical masks for primary care and gloves, medical/surgical masks and respirators (hospitals only).
- Percentage of facilities with available oxygen (primary care and hospitals).
- Percentage of facilities with a functioning invasive and/or non-invasive ventilator (hospitals only).
- Percentage of hospitals with onsite rapid diagnostic tests (RDT) and/or polymerase chain reaction (PCR) tests for COVID-19 diagnosis (hospitals only).
- Average availability of essential tracer diagnostics in facilities (average percentage of tracer items available) Items include tracer diagnostics to test for blood glucose, urine glucose, urine protein, pregnancy, HIV, tuberculosis, hemoglobin, and bloodtype as relevant (primary care only).
- Average availability of tracer therapeutics in facilities (average percentage of tracer items available) Items include tracer therapeutics to treat COVID-19 (hospitals only) and other essential health services (primary care only; see Annex 3 for complete list).

The overall average is calculated as the unweighted arithmetic mean of the countries with existing data.

As part of the broader approach, guidance was also provided on using routine data to monitor the effects of COVID-19 on essential health services (51). As such, efforts were made to use facility and community survey findings together with RHIS data to provide a more comprehensive picture of the supply-of and demand-for frontline health services. These findings are not presented in this paper, and will be published in forthcoming reports.

## Results

Three rounds of the national level key informant pulse survey during 2020–2021 demonstrated the sustained impact of the pandemic on health systems and essential health services over time. Respectively, 87% (187 of 216), 63% (136 of 216), and 59% (132 of 223) of surveyed countries, territories and areas^4^ responded to the first, second and third rounds of the pulse survey. The number of countries receiving the survey changed between rounds due to increased requests from WHO regions to include additional non-Member State territories and/or areas in the survey as well as requests from countries to submit multiple subnational responses when an aggregate national response was felt to be insufficient. In total 97 countries responded to at least one survey section in all three rounds. The time interval between the close of data collection and presentation of preliminary results to countries and partners was ~1 month.

The response rate varied by survey sections and by round [in round 3, response rate varied from 40 to 64% of countries where the area was relevant (i.e., malaria and NTDs survey sections were not asked in all countries)]. Additionally, responses of “Do not know” or “Not applicable” were not counted in the denominators. Response rate also varied by WHO regions. Overall, response rates in round 3 varied from 26% of countries in the Western Pacific region that responded to at least one survey section to 90% of countries in the Africa region that responded to at least one survey section. In round 2, response rates ranged from 43% in the European region to 95% in the Eastern Mediterranean region. In round 1 response rate ranged from 63% in the Americas region to 100% in both the Eastern Mediterranean and Southeast Asia regions.

**Figure 1:**
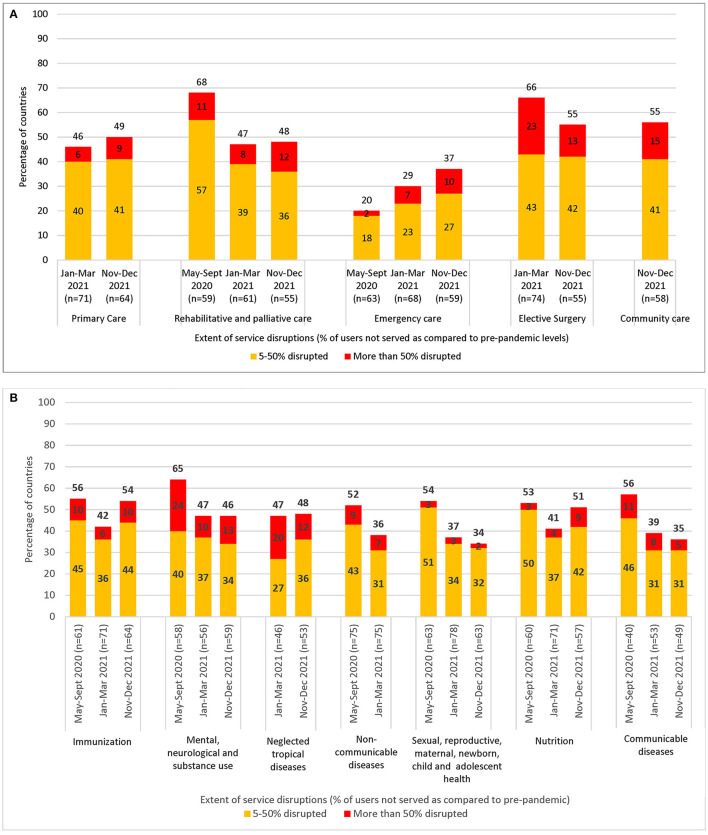
**(A)** Comparison of disruptions by service delivery setting in 97 countries responding to all three WHO pulse survey rounds: May–September 2020 (round 1), January–March 2021 (round 2), and November–December 2021 (round 3). Primary care services and elective surgeries were not included in me first May–September 2020 survey round. Community care services were not included in the first and second May–September 2020 and January–March 2021 survey rounds. As such, relevant service disruptions for these time periods are not presented. Each survey examined the situation in countries during a specific period of time. For rounds 1 and 2, the results refer to the period 3 months prior to survey and 6 months prior to survey response for round 3. **(B)** Comparison of disruptions by condition- or programme-specific tracer service area in 97 countries responding to all three WHO pulse survey rounds: May–September 2020 (round 1), January–March 2021 (round 2), and November–December 2021 (round 3). Neglected tropical diseases were not included in one first May–September 2020 survey round. Non-communicational diseases were not included in the second November–December 2021 survey round as a separate 2021 WHO NCD Country capacity survey was completed during a similar time period asking similar questions on disruptions. However, the methodology differed and consequently was not comparable for inclusion in terms of analysis trends. As such, relevant service disruptions for these time periods are not presented.

Findings from the most recent survey revealed that as of December 2021, 92% of responding countries across all income levels and regions were still reporting persisting disruptions to services. Disruptions were reported in all service delivery settings, with primary care and community care among the most affected, showing that many people were still missing out on essential first-contact care. Significant disruptions were also noted for elective surgeries and emergency care, especially critical for people with urgent health needs (Figure 1A). Moreover, disruptions were reported across all major condition- or programme-specific tracer health areas (Figure 1B) (30).

At the same time, 89 of 98^5^ (91%) countries reported at least one major health system bottleneck in round 3 of the pulse survey to scaling up access to COVID-19 therapeutics (83% of countries), COVID-19 diagnostics and testing (78% of countries), COVID-19 vaccination (74% of countries), and PPE (65% of countries). The most frequently reported bottlenecks included health workforce challenges, shortages in supplies and equipment, and demand-side challenges (most notably for COVID-19 vaccination).

**Figure 2:**
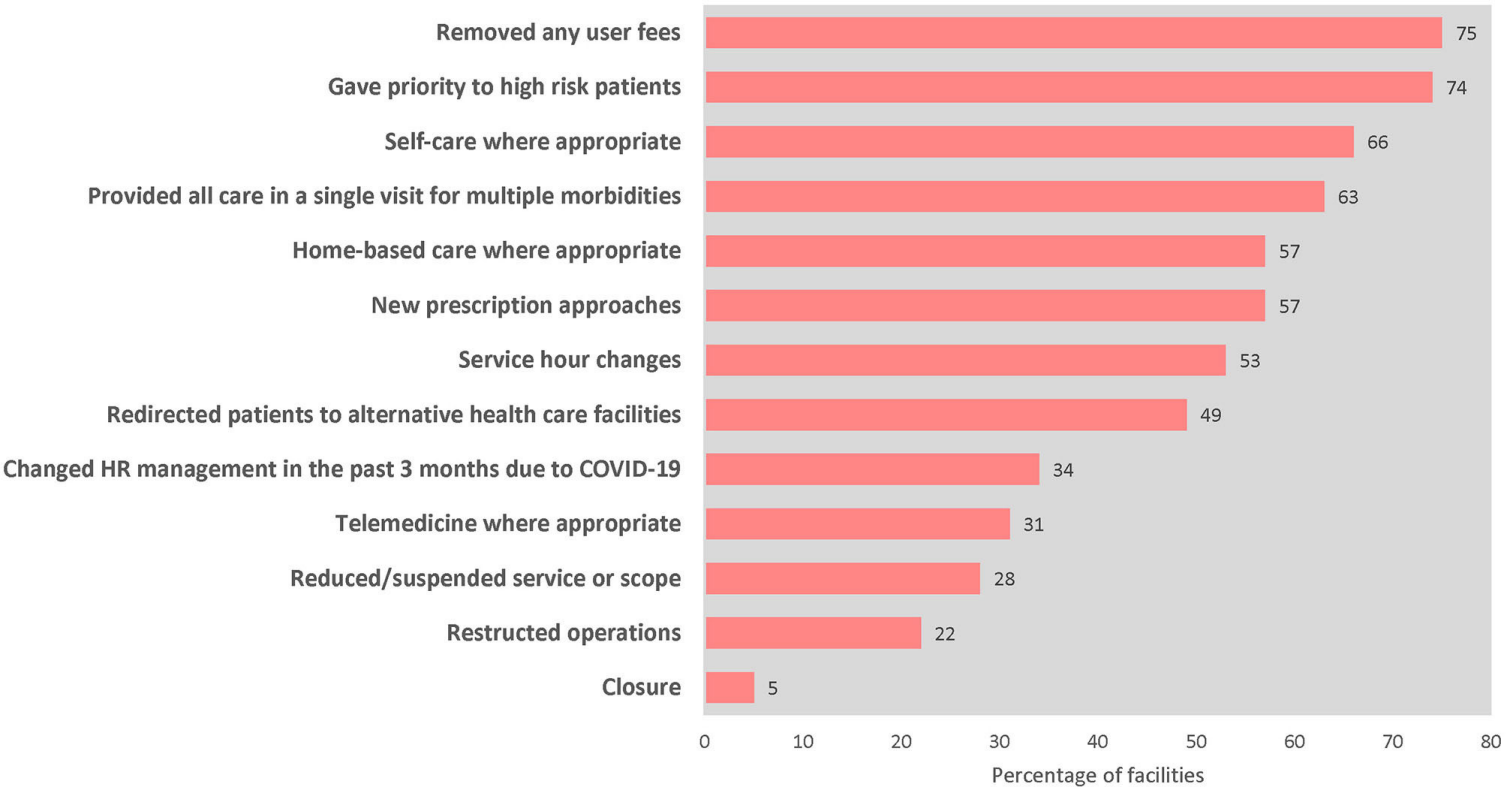
Percentage of primary care facilities (*n* = 2,377, 21 countries) that employed mitigation strategies to overcome service disruptions at the time of assessment (January 2021–March 2022).

All countries (*n* = 98; see text footnote 5) reported using different strategies and innovations to overcome challenges, including improving access to essential medicines and health products, health workforce mitigation measures, service delivery modifications, and pursuing different community engagement and health financing strategies (Figure 2).

Comprehensive pulse survey findings are published on WHO's website (28–30)^6^.

Health facility and community surveys on frontline health service capacities reflected similar challenges based on more granular level data. Facility managers and community representatives in 22^7^ countries reported varying levels of disruptions across service delivery settings, including to first-contact services. On average, almost 60% of primary care settings reported decreases in outpatient service volumes. Additionally, almost half of facilities reported scaled back outreach services. Disruptions were most often due to decreased demand, limited health system resources (e.g., health workers or essential health products), or intentional modifications to scale back services during COVID-19 outbreaks. Other facilities experienced disruptive surges in service volumes due to targeted campaigns and community communications to catch up on service backlogs.

Surveys with community representatives validated the notion that barriers to care had increased even further due to COVID-19 from the demand-side perspective. On average, over two-thirds of community representatives reported that the pandemic had moderately or severely affected people's access to care. Almost 90% also reported that people in their community had faced at least one unmet essential health need during the pandemic.

Facilities also reported shortages in health system resources needed to support the safe provision of care for both COVID-19 and other essential health services. Capacities for health worker protection were reported as problematic across all settings, with an average of only 49% of hospitals and 57% of primary care settings able to provide all tracer PPE items to all staff to protect them from infection (Table 1). Additionally, an average of 9% and 8% of clinical staff in hospitals and primary care, respectively, were affected by COVID-19 infection in the 3 months preceding the assessment. This is particularly concerning in the subset of 9 countries that are also on WHO's 2020 health workforce support and safeguard list, a list that identifies countries with health workforce availability of less than the global median of 48.6 per 10,000 population (52). In these settings, any additional restriction to health workforce availability, such as due to COVID-19 infection, could have detrimental effects on service delivery and outcomes.

**Table 1:** Availability of essential COVID-19 tools and other essential health products in hospitals (*n* = 498, 18 countries) and primary care facilities (*n* = 2,377, 21 countries) at the time of assessment, by country (January 2021–March 2022).

	**Hospitals**					**Primary care**			
	**% of facilities with PPE for all staff**	**% of facilities with onsite RDT and/or PCR for COVID-19 diagnosis**	**Average availability of therapeutics to treat COVID-19**	**% of facilities with functioning invasive and/or non-invasive ventilators**	**% of facilities with available oxygen**	**% of facilities with PPE for all staff**	**Average availability of essential diagnostics** ^a^	**Average availability of essential therapeutics** ^b^	**% of facilities with available oxygen**
Burundi	31	80	64	69	80	47	89	44	No data
Cameroon	18	62	63	55	75	40	75	65	No data
Chad	33	33	58	67	100	36	47	46	18
Congo	21	55	50	58	48	26	48	35	No data
Ghana	37	55	68	63	97	60	34	49	57
Kenya	21	53	59	96	99	12	60	55	14
Mali	36	0	74	100	100	57	80	55	29
Namibia	67	54	87	74	95	67	42	74	70
Senegal	36	100	63	93	93	60	95	71	51
Seychelles	No data	No data	No data	No data	No data	90	41	58	74
Zambia	56	96	68	64	100	47	72	58	32
Paraguay	95	95	87	100	100	25	No data	44	64
Peru	69	92	88	83	100	86	71	68	96
St. Lucia	No data	100	93	100	100	79	59	66	93
St. Vincent	100	100	80	100	100	71	44	62	93
Suriname	11	33	61	89	89	41	No data	No data	No data
Afghanistan	80	40	80	100	100	73	49	67	91
Libya	40	50	61	100	80	28	34	27	73
Yemen	87	87	59	100	91	68	65	51	80
Moldova	No data	No data	No data	No data	No data	99	65	75	78
Ukraine	No data	No data	No data	No data	No data	88	73	63	35
**Average**	**49**	**66**	**70**	**84**	**92**	**57**	**60**	**57**	**62**
**91% or more**	**80–90%**	**65–79%**	**50–64%**	**Less than 50%**	**No data**				
									

a Diagnostics for blood glucose, urine glucose, urine protein, pregnancy, HIV, TB, HBG, and bloodtype (as appropriate for facility type).

b See Annex 3 for therapeutics list.

Table 1 highlights shortages in other essential health product availability reported by hospitals in 18 countries and primary care settings in 21 countries. In terms of availability of health products for COVID-19 services, an average of 66% of hospitals reported availability of diagnostics for on-site COVID-19 testing. On average, hospitals only had about 70% of the tracer therapeutics for COVID-19 treatment available. In general, primary care settings showed even lower availability of essential health products. The average availability of tracer diagnostics and therapeutics for tracer essential health services in facilities was 60 and 57%, respectively. While a higher average of 92% of hospitals reported availability of oxygen, only 62% of lower-level facilities reported oxygen availability. These gaps bear implications not only in terms of COVID-19 case management, but also for the delivery of other routine and emergency essential health services.

**Figure 3:**
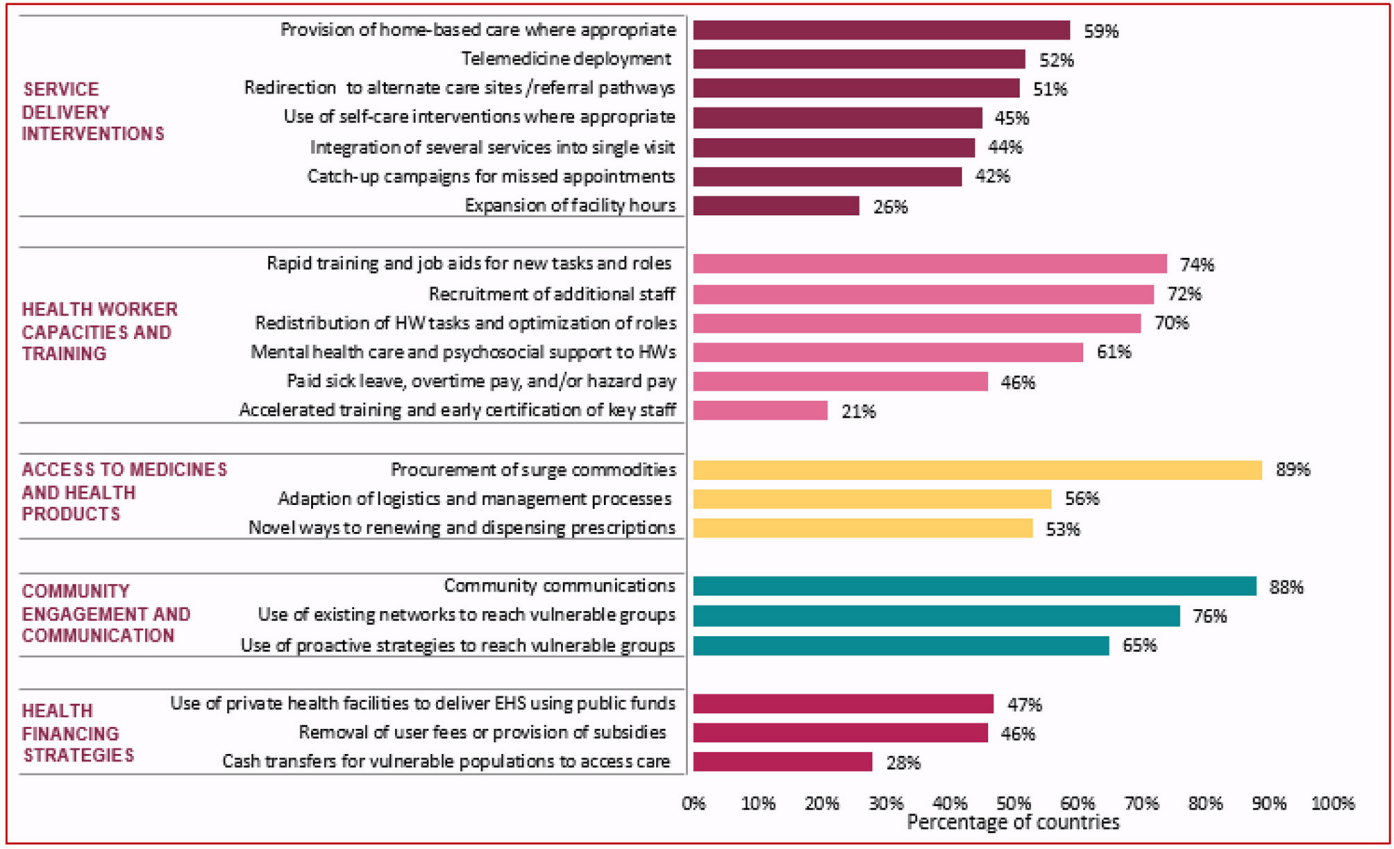
Percentage of countries implementing mitigation and recovery actions, November–December 2021 (*n* = 98). Examples of community communications included: communications to inform communities of changes to service delivery in the COVID-19 context communications to address misinformation and community fears of infection, targeted outreach where service utilization had declined, and the establishment of hotlines or community radios. HW, health workers; EHS, essential health services.

All facilities adapted to these health system restraints and demand-side challenges by employing mitigation strategies (Figure 3). Reported mitigation strategies include the removal of user fees, adaptations to facility service hours, and innovative service delivery adaptations (such as promotion of telemedicine or home-based care) to improve access to care. Many facilities also adopted changes in human resources management to improve availability of health workers.

## Discussion

In the wake of the pandemic, there has been a major recognition of the need to more intentionally leverage and design health system investments and interventions to make joint progress toward health security and universal health coverage based on a primary health care approach (53, 54). Central to this, is the use of evidence to strengthen health service readiness, resilience and responsiveness, with an emphasis on reducing barriers to care for the most vulnerable populations (55).

Disruptions are of particular concern in countries where service coverage was already limited before the pandemic, including fragile, conflict, and vulnerable settings. In this light, the methods presented in this paper contributed to country response during the COVID-19 pandemic, while building toward more resilient and sustainable data systems for future health crises.

Pulse survey country findings were used in various policy briefs (56), public health conferences (57), webinars, and country policy dialogues or roundtable discussions (often in triangulation with other country data from RHIS, surveillance systems, facility surveys, and household surveys). These mechanisms helped to synthesize and communicate findings to identify critical bottlenecks, trigger more in-depth assessments as needed, and ultimately inform the development of operational action plans to mitigate disruptions and address service backlogs. At the same time, the mechanisms contributed to the development of longer-term health service recovery and resilience building strategies for the future in many countries. In addition to country use, the pulse survey also filled important data gaps for monitoring global progress of multiple response-related plans, including WHO's COVID-19 Strategic Preparedness and Response Plan (58, 59), and the Global Humanitarian Response Plan for COVID-19 (60).

Findings from the facility and community surveys rapidly provided near-to-real-time data on what was happening at frontline health services in terms of the impact of COVID-19 on health care provision. Broadly, countries used the findings to inform decision-making and the development of action plans from national to facility levels for restoring services and strengthening facility capacities to respond to demands for both COVID-19 as well as other essential health services. When implemented regularly, the surveys allowed countries to alert changes in service capacities and track trends in recovery over time. Examples of key actions that countries have taken based on the survey findings include: prioritization of PPE access for all health staff in Kenya [(61), unpublished reports]^8^; investments to improve equitable access to oxygen and ventilators in hospitals in Ghana [(62), unpublished reports]^9^; the establishment of new COVID-19 testing and treatment centers in areas of need in Zambia (unpublished reports)^10^; and activities to empower community health workers to engage more regularly with community members to address demand-side challenges in Afghanistan (unpublished reports)^11^.

Findings from the national, facility and community surveys were also integrated into the Global COVID-19 Access Tracker dashboard (63) and other global dashboards for tracking service disruptions (64). These dashboards have been used to inform country situation analyses and trigger partner investments for country support and targeted delivery of essential tools and supplies, most notably in the context of the Access to COVID-19 Tools Accelerator (27). Partners have also made use of certain of components of the tools to assess disruptions and guide investments for specific programme areas during the pandemic, including for maternal, newborn, child and adolescent health, HIV, tuberculosis, and malaria (65–68).

### Successes and lessons learned

Limitations of the national, facility and community surveys should be considered. Firstly, responses provided by key informants reflect self-assessment, which may be prone to bias and lacks validation. For the pulse survey in particular, response rates reduced with each round, suggesting potential survey fatigue or reducing information gains for countries at different points of recovery. Furthermore, with a national focus, countries with considerable subnational variation may find the information less helpful. Dissemination of findings also presented difficulties in the rapidly evolving outbreak context, where traditional modes of data dissemination were not possible (e.g., in-person country workshops and policy dialogues).

Nonetheless, the methods and tools helped to fill critical gaps by generating actionable and dynamic data that was previously unavailable from global to local levels. The **pulse survey** offered one of the few globally comparable sources of country data on health service disruptions and system bottlenecks caused by COVID-19. In countries, the approach mitigated reporting burden and fostered cross-programme discussions, by offering one coordinated and comprehensive tool to assess different service areas. Moreover, to the extent that validation has been possible, the findings have echoed other studies that found consistent but variable impacts on essential health services across health domains (4, 5, 69–73).

The frontline health service capacity surveys provided dynamic supply- and demand-side data on frontline health service delivery and capacities that was previously missing through routine country monitoring systems. Countries disseminated findings through virtual meetings and online communications to guide actions and investments to mitigate the potential impact of COVID-19 on health outcomes in the long-term. The online, phone-based format also allowed for rapid, safe and contact-less data collection during the COVID-19 context, using fewer resources and logistical requirements compared to other in-person assessments. Moreover, the streamlined implementation support materials enabled rapid turnaround of results.

Implementation was most successful when strong country leadership and ownership was present, when country capacities for tracking health service readiness and resilience were strengthened, and when methods and tools were integrated into broader national and local operational planning processes.

In this way, the approach successfully provided a low-cost, action-oriented method to collect critical operational information from national to local levels on health service readiness, resilience, and responsiveness during the COVID-19 pandemic, and highlighted the importance of building more responsive and resilient country monitoring systems for the future.

### Implications for the future

Now, as countries review, recover and transform health systems to make them more robust and resilient in the event of future shocks, countries have expressed interest to more sustainably institutionalize core components of the methods and tools into routine country data systems.

The surveys are being evaluated further to inform their potential integration into regular country data systems. This includes reviewing the consistency of findings with other country data sources, particularly from RHIS. Publication of these results is forthcoming. To complement the breadth of these survey results, it may also be valuable to conduct in-depth studies to assess the impact of COVID-19 on essential health services using inferential statistics, and to assess the linkages between health service readiness and health impact more closely (9, 74, 75). Further testing on best practices for integrating the methods and tools into existing country data systems and aligning them with broader national and local policy and planning processes and dialogues will also be helpful.

Notwithstanding the need for further validation and testing, the rapid key informant tools and methods have successfully built country capacities, filled critical information gaps using minimal resources, and improved the use of data to inform actions, investments, and response from local to global levels in the pandemic context. They offer a promising approach to guide longer-term recovery efforts, to bolster routine health services monitoring systems, and to ultimately serve as health services surveillance and alert functions for future health crises.

## Data availability statement

The data is not immediately shareable as there are other publications underway using the same datasets. In addition, country approvals are required before the data are shared publicly. Requests to access the datasets should be directed to KO'N, oneillk@who.int and BR-M, rivasb@who.int.

## Author contributions

KO'N, HKa, BD, AR, HS, AT, and TZ: conceptualization and technical oversight. DH, BR-M, KV, RT-O, LN, MT, HKi, NH, DA, HD, and FT: coordination of survey implementation. CT, KV, AB, and BR-M: data curation, analysis, and synthesis. KO'N, BR-M, DH, KV, CT, and AB: writing. All authors contributed to the article and approved the submitted version.

## Footnotes

^1^The Access to COVID-19 Tools Accelerator (ACT-A) is a global partnership that was formed to end the pandemic as a global emergency. This work particularly contributed to the ACT-A Health Systems and Response Connector in the partnership.

^2^WHO has six regions as described above. The list of countries by region can be found at: https://www.who.int/countries.

^3^Given the country-tailored approach, the exact methodology and/or sampling approach may vary. Countries could choose to adapt the data collection modality (such as to in-person or online self-reporting), data collection platform, sampling approach, or frequency as relevant to country context, resources, priorities and need.

^4^From here on, the use of the term “countries” will refer to countries, territories and areas for the pulse survey results.

^5^Results for health systems bottlenecks and mitigation strategies are only presented for round 3. Ninety-eight countries responded to questions in these areas in round 3.

^6^Response rates and findings may vary slightly compared to those included in the published global reports because some country responses were received after report publication.

^7^One country, Zimbabwe, only conducted the community survey. Therefore, results at the health facility level are only presented for 21 countries.

^8^Unpublished reports include: Ministry of Health, Republic of Kenya. Readiness for COVID-19 Response and Continuity of Essential Health Services in Health Facilities and Communities February 2021; Ministry of Health, Republic of Kenya. Readiness for COVID-19 Response and Continuity of Essential Health Services in Health Facilities April 2021; Ministry of Health, Republic of Kenya. Trends in COVID-19 Response and Continuity of Essential Health Services in Health Facilities and Communities December 2021; Ministry of Health, Republic of Kenya. Readiness for COVID-19 Response and Continuity of Essential Health Services in Health Facilities and Communities—Experience from Kenya readiness assessments in the context of the COVID-19 pandemic May–June 2022 (presentation).

^9^Unpublished reports include: Ministry of Health, Republic of Ghana. Health service readiness for COVID-19 response and continuity of essential health services in health facilities. Results from the first Ghana readiness survey, June 2021; Ghana Health Services. Innovations, Developments, Challenges, and Lessons learned for monitoring frontline health services utilization and readiness during COVID-19 in Ghana (presentation).

^10^Unpublished reports include: Zambian Ministry of Health. Zambia Assessment on Service Readiness and Capacities in the context of COVID-19, June 2021; Zambian Ministry of Health. Monitoring frontline health services utilization and readiness during COVID-19 in Zambia, 2022 (presentation).

^11^Unpublished reports include: World Health Organization Country Office for Afghanistan. Maintaining the Essential Health Services in the context of the COVID-19 pandemic in Afghanistan (January–March 2022) Study report, World Health Organization Country Office for Afghanistan. Frontline Service Readiness Assessment—Afghanistan (January–March 2022) (presentation).
