# Supplementary material for: Assessing capacities and resilience of health services during the COVID-19 pandemic: Lessons learned from use of rapid key informant surveys
Source: Front Public Health. 2023 Feb 13;11:1102507. doi: 10.3389/fpubh.2023.1102507 (PMC9969144; doi:10.3389/fpubh.2023.1102507)
Supplement: Supplementary file 1 [file Table_1.DOCX]

| **Country** | **Most recent round** | **Continuity of essential health services module**  (mainly conducted in primary care facilities or other lower-level facilities) | | **COVID-19 case management capacities module**  (mainly conducted in hospitals or other higher-level facilities) | | **Community perceptions, needs and demands module**  (mainly conducted with community health workers or other community representatives) | |
| --- | --- | --- | --- | --- | --- | --- | --- |
|  |  | **Rounds (n)** | **Facilities in most recent round (n)** | **Rounds (n)** | **Facilities in most recent round (n)** | **Rounds (n)** | **Communities in most recent round (n)** |
| **African region** | | | | | | | |
| Burundi | Dec. 2021 | 1 | 117 | 1 | 65 | 1 | 45 |
| Cameroon | Apr. 2021 | 1 | 186 | 1 | 60 | 1 | 34 |
| Chad | Feb. 2022 | 1 | 191 | 1 | 3 | 1 | 109 |
| Congo | Dec. 2021 | 1 | 153 | 1 | 33 | N/A | N/A |
| Ghana | Nov. 2021 | 2 | 146 | 2 | 38 | 2 | 212 |
| Kenya | Nov. 2021 | 3 | 93 | 3 | 68 | 3 | 99 |
| Mali | Aug. 2021 | 1 | 212 | 1 | 14 | 1 | 73 |
| Namibia | Aug. 2021 | 1 | 116 | 1 | 43 | N/A | N/A |
| Senegal | Jan. 2021 | 1 | 68 | 1 | 14 | 1 | 74 |
| Seychelles | Dec. 2021 | 1 | 34 | N/A | N/A | N/A | N/A |
| Zambia | Dec. 2021 | 2 | 248 | 2 | 55 | 2 | 49 |
| Zimbabwe | Nov. 2021 | N/A | N/A | N/A | N/A | 1 | 295 |
| **Americas region** | | | | | | | |
| Paraguay | Nov. 2021 | 1 | 70 | 1 | 20 | 1 | 40 |
| Peru | Oct. 2021 | 1 | 112 | 1 | 36 | 1 | 36 |
| St. Lucia | Mar. 2022 | 1 | 14 | 1 | 1 | 1 | 15 |
| St. Vincent | Mar. 2022 | 1 | 15 | 1 | 1 | 1 | 5 |
| Suriname | Sept. 2021 | 1 | 27 | 1 | 9 | 1 | 20 |
| **Eastern Mediterranean region** | | | | | | | |
| Afghanistan | Feb. 2022 | 1 | 92 | 1 | 5 | 1 | 25 |
| Libya | Mar. 2022 | 1 | 40 | 1 | 10 | 1 | 50 |
| Yemen | Mar. 2022 | 1 | 94 | 1 | 23 | 1 | 96 |
| **European region** | | | | | | | |
| Moldova | Sept. 2021 | 1 | 99 | N/A | N/A | N/A | N/A |
| Ukraine | Dec. 2021 | 2 | 250 | N/A | N/A | N/A | N/A |
| **Total** |  |  | **2377** |  | **748** |  | **1427** |

**ANNEX 1. Countries that implemented frontline health service capacity surveys in facilities or communities between December 2020 – June 2021: Assessment rounds and the number of facilities or community representatives included in the most recent survey sample^[[1]](#footnote-2)^**

1. The above table includes only countries that implemented facility and community surveys and opted into WHO’s data sharing agreement [↑](#footnote-ref-2)
