# Supplementary material for: Assessing capacities and resilience of health services during the COVID-19 pandemic: Lessons learned from use of rapid key informant surveys
Source: Front Public Health. 2023 Feb 13;11:1102507. doi: 10.3389/fpubh.2023.1102507 (PMC9969144; doi:10.3389/fpubh.2023.1102507)
Supplement: Supplementary file 2 [file Table_2.DOCX]

**ANNEX 2. Purpose and main contents of core assessment modules from WHO’s suite of frontline health service capacity assessments**

| **#** | **Assessment tool** | **Purpose** | **Respondents** | **Contents** |
| --- | --- | --- | --- | --- |
| **FACILITY ASSESSMENT MODULES: HOSPITALS AND PRIMARY CARE** | | | | |
| 1 | **COVID-19 case management capacities: Diagnostics, therapeutics, and vaccine readiness** (37) | Assess present and surge capacities for the treatment of COVID-19 in health facilities, with a focus on availability of COVID-19 therapeutics, diagnostics, oxygen, PPE, vaccines, and vaccine readiness | Facility managers in hospitals and COVID-19 treatment centres | 1. Health facility identification and description 2. Staffing and incident management support team 3. Case management and bed capacity for COVID-19 patients 4. Selected medicines and supplies for COVID-19 case management 5. Personal protective equipment and infection prevention and control 6. COVID-19 laboratory diagnostics 7. Medical equipment for diagnosis, patient monitoring and case management 8. General vaccine readiness 9. COVID-19 vaccine readiness |
| 2 | **Continuity of essential health services** (38) | Assess health facility and health workforce capacities to maintain the safe provision of essential health services | Facility managers in primary care settings and referral hospitals | 1. Staffing 2. Financial management 3. Service delivery and utilization 4. COVID-19 infection prevention and control and personal protective equipment 5. Management of suspected and confirmed COVID-19 cases in primary care centres 6. Availability of selected tracer therapeutics (optional) 7. Availability of diagnostics (optional) 8. General vaccine readiness (optional) 9. COVID-19 vaccine readiness (optional) 10. Facility infrastructure (optional) |
| **COMMUNITY ASSESSMENT MODULE** | | | | |
| 3 | **Community needs, perceptions and demand – community assessment tool** (39) | Assess community needs, changes in care-seeking behaviours, and barriers to accessing care in the COVID-19 context | Community representatives (e.g. community health workers, community leaders, etc.) | 1. Need for and use of essential health services in communities 2. Barriers to seeking essential health services in communities 3. Attitudes towards COVID-19 vaccine 4. Community assets and vulnerabilities 5. Barriers to delivery of community-based services (if applicable) 6. Follow-up consent and interview result |
