# Supplementary material for: Assessing capacities and resilience of health services during the COVID-19 pandemic: Lessons learned from use of rapid key informant surveys
Source: Front Public Health. 2023 Feb 13;11:1102507. doi: 10.3389/fpubh.2023.1102507 (PMC9969144; doi:10.3389/fpubh.2023.1102507)
Supplement: Supplementary file 3 [file Table_3.DOCX]

**ANNEX 3. Essential list of tracer therapeutics assessed in facility surveys**

**Selected therapeutics for COVID-19 case management in hospitals**

Rubbing alcohol (>70% alcohol by volume)

Chlorine High Test Hypochlorite (HTH) 70%

Paracetamol (for oral administration)

Ampicillin (injectable)

Ceftriaxone (injectable)

Azithromycin (for oral administration)

Dexamethasone (injectable)/ corticosteroids

Tocilizumab /IL-6 inhibitors (injectable)

Thromboprophylaxis: Heparin, Low molecular weight heparin (injectable)

Rocuronium (injectable) or other neuromuscular blocker

Morphine (injectable) or other opiate Morphine

Haloperidol (injectable)

Epinephrine or noradrenaline (injectable)

Intravenous fluids: normal saline or Ringer’s lactate / balanced crystalloids

Oxygen

**Selected essential therapeutics in primary care**

Salbutamol

Metformin

Hydrochlorothiazide

Paracetamol

Carbamazepine

Amoxicillin

Ethinylestradiol + levonorgestrel (or alternative combined oral contraceptive)

Oxytocin

Magnesium sulfate

Heparin

Hydrocortisone or dexamethasone

Epinephrine

Artemether + lumefantrine (or other artemether combination medicine)

Efavirenz + emtricitabine + tenofovir disoproxil fumarate

Isoniazid + pyrazinamide + rifampicin

Intravenous (IV) fluids (normal saline or Ringer’s lactate)

Oxygen
